# Characteristics of Chest CT Images in Patients With COVID-19 Pneumonia in London, UK

**DOI:** 10.7759/cureus.10289

**Published:** 2020-09-07

**Authors:** Emre Pakdemirli, Uday Mandalia, Sherif Monib

**Affiliations:** 1 Radiology, West Hertfordshire Hospitals NHS Trust–St Albans City Hospital, St Albans, GBR; 2 Radiology, West Hertfordshire Hospitals NHS Trust–Watford General Hospital, Watford, GBR; 3 Breast Surgery, West Hertfordshire Hospitals NHS Trust–Watford General Hospital, Watford, GBR

**Keywords:** novel 2019 coronavirus, covid-19, chest ct, rt-pcr, viral pneumonia

## Abstract

Background and objective

Novel coronavirus 2019 (COVID-19) outbreak was first reported in Wuhan, Hubei Province in China in December 2019; it has then spread quickly and exponentially beyond the Chinese borders and is now regarded as a global pandemic. We aimed to evaluate the chest CT radiological characteristics and lesion distribution patterns in patients of COVID-19 pneumonia in London, UK.

Methods

We performed a retrospective study and reviewed data of patients with clinically suspected COVID-19 who underwent chest CT between February 1 and May 5, 2020. All patients underwent the reverse transcription-polymerase chain reaction (RT-PCR) test. Lung lesion characteristics and distribution patterns were evaluated by two radiologists. Fisher’s exact test was used for statistical analysis, and a p-value of <0.05 was considered statistically significant.

Results

A total of 18 patients (nine men and nine women) were analyzed. All of them had bilateral patchy lesions in the chest CT images. There was no correlation between the severity score and mortality (p=0.790). The distinctive CT features included ground-glass opacity (GGO) and consolidative patchy amorphous lesions, bilateral posterior and peripheral multi-lobar lung involvement, pleural effusions, subpleural fibrotic lines, subpleural sparing, vascular engorgement, occasional crazy paving, occasional mediastinal lymphadenopathy, pleural thickening, lack of cavitation, and absence of reverse halo (atoll) signs.

Conclusion

CT can facilitate the diagnosis of COVID-19 pneumonia. Our UK cohort showed slight variations compared with previously reported Asian and continental European cases with respect to chest CT images.

## Introduction

Novel coronavirus disease 2019 (COVID-19) outbreak emerged in Wuhan in Hubei province, China, in December 2019 [1-6]. It has spread quickly and exponentially outside China since January 2020. The World Health Organization (WHO) has considered the risk of COVID-19 to be very high globally and declared it as a pandemic disease in February 2020 [7]. The US is the country with the highest COVID-19 mortality rate in the world so far; the UK ranks second in this list at the time of writing this report in May 2020.

It is hypothesized that COVID-19 originally spread from bats to humans, and was then rapidly transmitted from human to human. There is no established or approved treatment for COVID-19. Although the mortality rates are not high, the virus is potentially lethal. The real-time reverse transcription-polymerase chain reaction (RT-PCR) test is accepted as the gold standard in COVID-19 diagnosis. However, recent studies have emphasized the importance of chest CT examination in COVID-19 patients in light of reports of false-negative RT-PCR test results [8,9].

CT features and symptoms of COVID-19 show overlap with those of Middle East respiratory syndrome (MERS) and severe acute respiratory syndrome (SARS) [10]. The most common abnormalities observed in the chest CT are ground-glass opacity (GGO), consolidation, or both. The most common shapes are amorphous patchy, nodular, patchy-nodular, and rounded lesions. The most common location involved in the lungs is posterior, peripheral, and lower lobes. Other common associated findings can be halo sign, focal vascular engorgement, crazy paving, pleural thickening, pleural effusion, subpleural lines, air bronchograms, bronchial wall thickening, and bronchial distortion [2,5,6,9,10].

The majority of the current publications on COVID-19 CT features have been from China and continental Europe. To the best of our knowledge, no report from London, UK has been published at the time of writing this paper. Therefore, we performed a retrospective analysis of chest CT images in a small but diverse COVID-19 patient cohort in London, UK, with an emphasis on the associated laboratory findings. Chest X-Ray is the first-line radiologic investigation for diagnosis and monitoring the disease; hence, as a commonly accepted practice, CT chest scanning for COVID-19 pneumonia should be reserved for cases of equivocal chest X-Ray, initial negative RT-PCR test, and for monitoring the disease.

## Materials and methods

This retrospective study was registered on the Integrated Research Application System (IRAS), a national UK research portal (IRAS no.: 282408). Ethical approval was granted by the National Health Service (NHS) Health Research Authority (HRA) Research Ethical Committee. Patient informed consent was waived by the HRA.

Study participants

Patient data were obtained from the hospital radiology information system (CRIS) by searching the radiology reports and patient clinical portal with the keywords “COVID,” “coronavirus,” and “COVID-19” from February 1 to May 5, 2020.

The inclusion criteria for the study participants were as follows: 1) high clinical or initial radiological suspicion, e.g., fever, cough, shortness of breath, dependence on O_2_, or non-specific patchy shadowing on chest X-ray; 2) RT-PCR-positive and negative patients (after two consecutive RT-PCR testings, none of the patients were allowed to take tests more than twice in our hospital settings) with strong clinical suspicion who were treated and followed up as cases of COVID-19 pneumonia; 3) positive or equivocal X-ray findings for COVID-19 pneumonia; 4) patients between 18-100 years of age; 5) patients with complete records on chest CT images.

The exclusion criteria were as follows: 1) reports that contain the words “No COVID pneumonia,” “No evidence of novel coronavirus,” “No COVID-19,” or “No atypical pneumonia/pneumonic process”; 2) reports with a clinical details section that contains the words “? COVID” or reports in which the text body contains the words “No COVID.”

**Figure 1:**
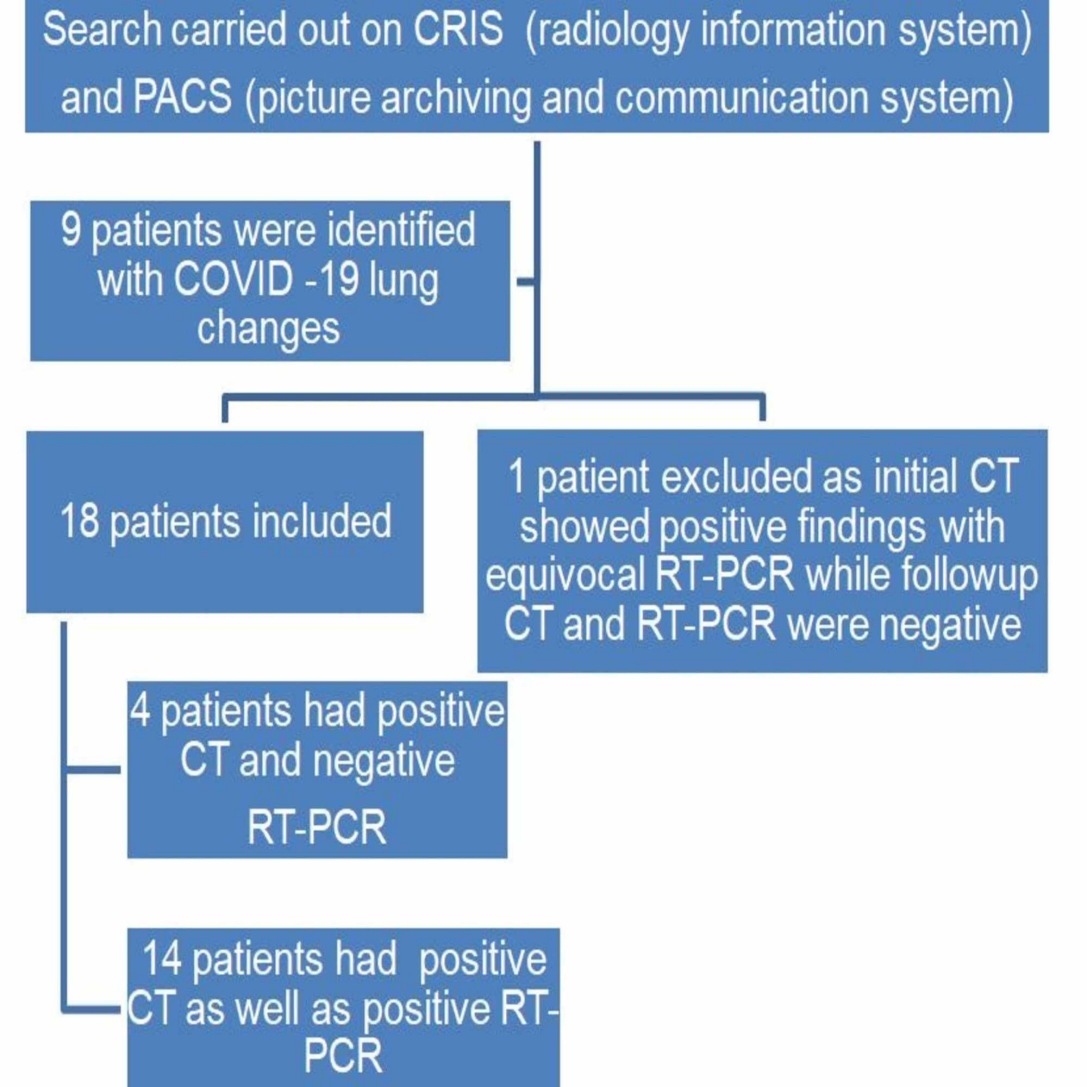
Study algorithm COVID-19: coronavirus disease 2019; CT: computed tomography; RT-PCR: reverse transcription-polymerase chain reaction

Ultimately, a total of 18 patients (median age: 57 years) who were considered positive for COVID-19 viral pneumonia on CT imaging were selected for the study (Figure 1). 

CT protocols

High-resolution CT (HRCT), CT of the chest, abdomen, and pelvis (CT-CAP), and/or CT pulmonary angiography (CTPA) were used for detecting pulmonary lesions. All images were obtained with a CT scan (GE Frontier; GE Healthcare, Chicago, IL) with patients in the supine position. Scans were performed with the following technical parameters: HRCT and CT-CAP: 120 kV, auto modulated mA, 0.625 slice thickness, 512×512 matrix; CTPA: 100 kV, auto modulated mA, 0.625 slice thickness, 512×512 matrix. Reconstructed images were also obtained and used for the current study.

Image analysis

**Figure 2:**
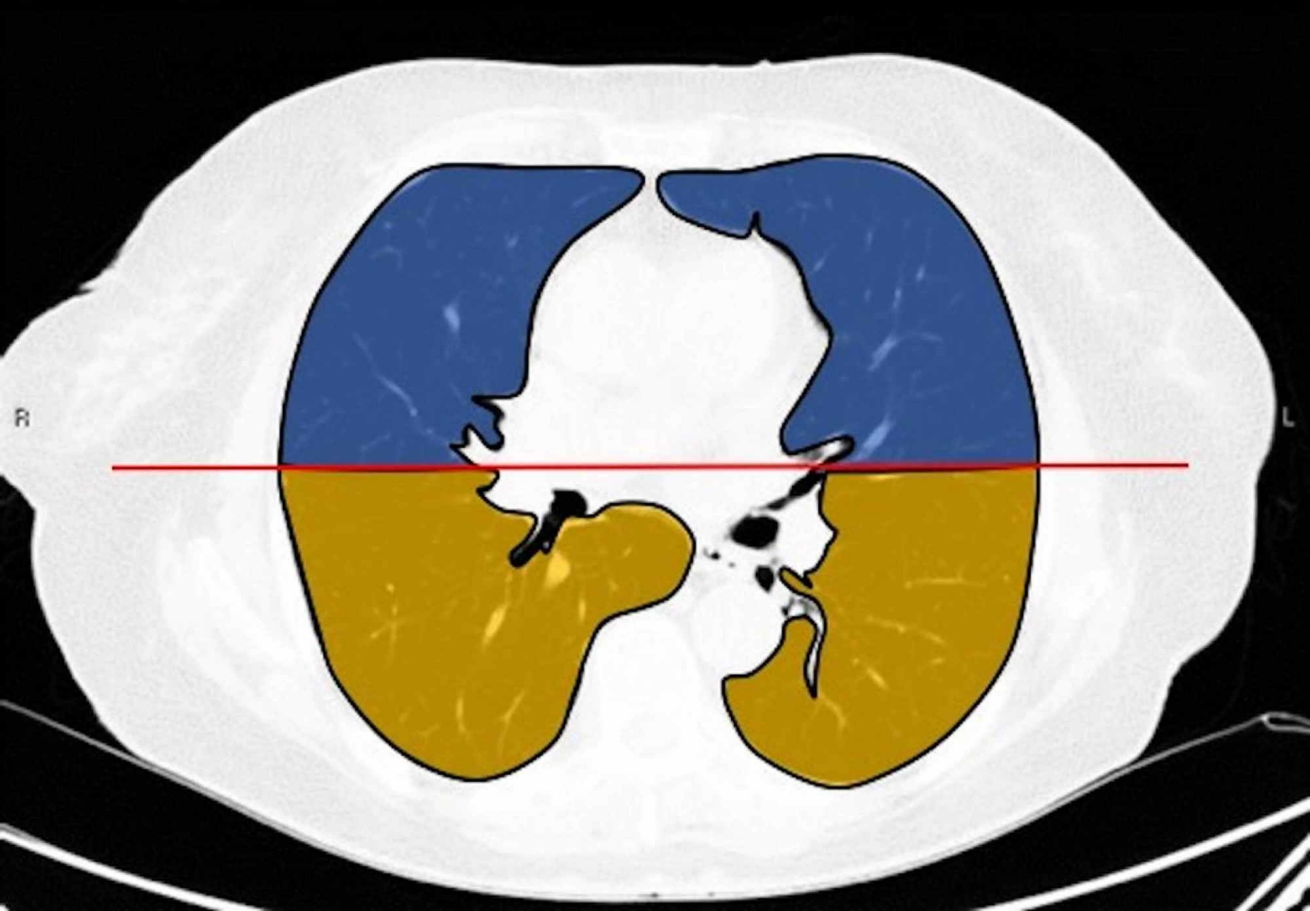
Division of lung fields in the axial plane Dividing lung fields equally into two axial planes on CT: blue represents the anterior half; brown represents the posterior half CT: computed tomography

Two board-certified radiologists (E.P. and U.M., with 25 and five years of experience, respectively) reviewed the chest CT images on a picture archiving and communication system (PACS, Carestream Health, Inc, Rochester, NY). Chest CT images were evaluated with both mediastinal (width: 350 HU, level: 40 HU) and lung (width: 1500 HU, level: -500 HU) window level settings. The two radiologists identified pulmonary lesions based on their density, shape, and margin. The locations of the lung lesions were recorded as lobar, axial, anterior, and posterior. Axial locations were categorized as central (inner two-thirds of the lung) or peripheral (outer one-third of the lung). Each lung was divided by an axial line into anterior and posterior halves (Figures 2, 7). The densities of the patchy-confluent lesions were classified as pure GGO, pure consolidation, or mixed. Nodular lesion densities were classified as pure GGO, solid, or partly solid. Margins were classified as well-defined or ill-defined. As most of the lesions were patchy, confluent, and ill-defined margins, lesion sizes were not assessed. Instead, a visual severity score, which was slightly modified from Pan et al., ranging from 0 to 4 for each individual lobes was calculated. Each of the five lung lobes was visually scored on a scale of 0 to 4 as follows: 0: no involvement; 1: less than 25% involvement; 2: 25-50% involvement; 3: 50-75% involvement; and 4: 75-100% involvement.

The total CT score was the sum of all individual five lobar scores ranging from 0 (no involvement) to 20 (maximum involvement).

Laboratory tests

All patients underwent nose-throat swab tests for RT-PCR (Xpert® Xpress SARS-CoV-2, Cepheid Inc, Sunnyvale, CA; FDA-endorsed) and film array, neutrophil, lymphocyte, C-reactive protein (CRP), and D-dimer tests.

Statistical analysis

The distributions of the lesion pattern and severity score proportions were analyzed. Fisher’s exact test (SPSS Statistics version 25; IBM, Armonk, NY) was applied to analyze the relationship between the severity score and death. A p-value of <0.05 was considered statistically significant.

## Results

A total of 18 patients (median age: 57) who were considered positive for COVID-19 viral pneumonia on CT imaging were selected. Of them, four had negative RT-PCR results and 14 had positive RT-PCR results (Figure 1). Patient demographics are summarized in Table 1. No gender predilection was encountered in our cohort (nine men and nine women). In a majority of cases (15 out of 18), symptoms were chest-related. The remaining cases presented with abdominal symptoms. One patient was diagnosed with small-bowel obstruction. Table 2 lays out clinical, laboratory, and radiological data for all patients.

**Table 1:** Demographic details of the patients *Mean age: 53.3 years; median age: 57 years

Age* (years)	Frequency	Gender (F/M, n)	Percentage
20–29	1	1/0	6%
30–39	2	0/2	11%
40–49	4	2/2	22%
50–59	2	0/2	11%
60–69	3	2/1	17%
70–79	4	2/2	22%
80–89	2	1/1	11%
Total	18	9/9	100%

**Table 2:** Clinical details, chest X-ray, laboratory findings, and patient outcomes RT-PCR: reverse transcription-polymerase chain reaction; CRP: C-reactive protein

Predominant clinical complaints	Number of cases	Percentage
Cough	8	44%
Fever	9	50%
Shortness of breath	7	39%
Abdominal pain	2	11%
Vomiting	1	6%
Pleuritic chest pain	2	11%
X-ray +	11	61%
X-ray −	2	11%
X-ray equivocal	4	22%
X-ray not performed	1	6%
RT-PCR +	13	67%
RT-PCR –	4	22%
RT-PCR not performed	1	6%
High CRP levels	18	100%
High D-dimer levels	5	28%
D-dimer levels not measured	13	72%
Mortality		
Alive	14	78%
Dead	4	22%

Chest image results

Of the 18 cases, 11 had positive X-ray examination results, four had equivocal bilateral ill-defined subtle X-ray changes, and one had negative X-ray results but with CT images consistent with COVID-19 pneumonia.

In the CT images, GGO plus consolidation was observed bilaterally in all 18 (100%) patients. No pure GGO or pure consolidation was encountered in our cohort. The predominant shape was amorphous (72%). Lesions showed no specific lobar predilection, but most lesions were localized posteriorly and peripherally.

Laboratory tests

Of the 18 patients with epidemiological, clinical, and radiological findings consistent with COVID-19 pneumonia, 14 had positive and four had negative RT-PCR test results. Film arrays were negative for the vast majority of cases, particularly for patients with negative RT-PCR test results, except for one case (film array was not performed for that particular patient). White blood cell counts were normal in 15 out of 18 patients and low in three out of 18 patients. Lymphocyte counts were low in 10 out of 18 cases. CRP levels were elevated in 17 of 18 cases. Of 18 patients, four had positive D-dimer test results, but no pulmonary embolus (PE) was encountered on CTPA.

The distinctive CT features in our cohort are summarized in Table 3, including GGO and consolidative patchy mostly amorphous (72%) lesions, bilateral posterior and peripheral multi-lobar lung involvement, pleural effusions, subpleural fibrotic lines, subpleural sparing, vascular engorgement, occasional crazy paving, occasional mediastinal lymphadenopathy, pleural thickening, lack of cavitation, and absence of reverse halo (atoll) signs.

Subpleural sparing, which was observed in this patient cohort, has never been reported before, whereas mediastinal lymphadenopathy has been reported occasionally in the literature in English [11]. Focal vascular engorgement, septal thickening, and a subpleural fibrotic line were seen frequently in our cohort, with frequencies of 83%, 72%, and 61%, respectively.

**Figure 3:**
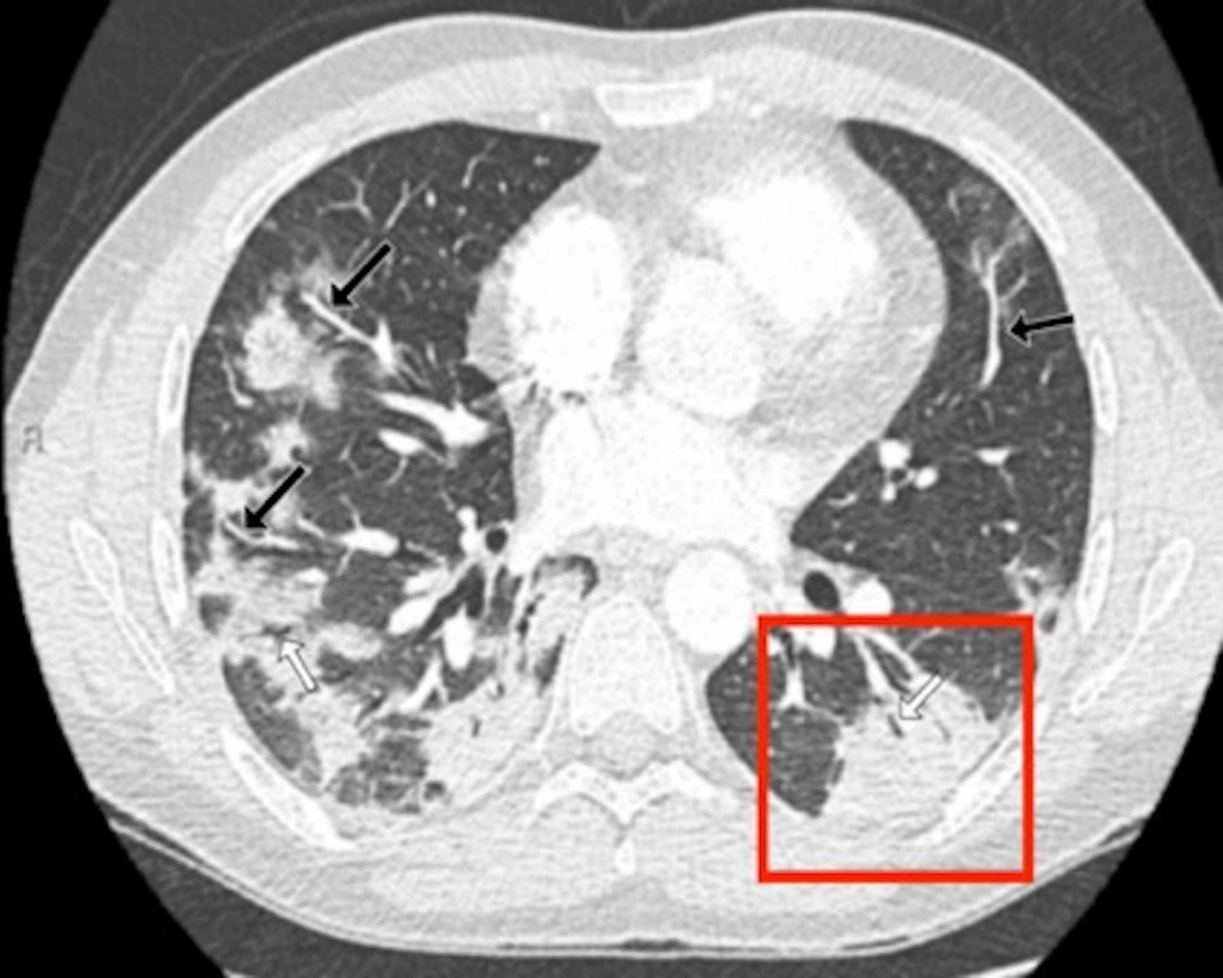
Chest CT findings of a 32-year-old male patient: axial CT section through lung bases A 32-year-old male patient presented with sudden-onset shortness of breath and chest pain after returning from abroad on a long-distance flight. A CT pulmonary angiogram was performed as a pulmonary embolus was suspected. His subsequent RT-PCR result was positive. An axial contrast-enhanced axial chest CT image demonstrated typical consolidative changes associated with COVID-19. The image showed bilateral large rounded consolidations predominantly posteriorly and within the periphery of both lower lobes. The largest lesion lay in the left lower lobe (red box). There was evidence of vascular engorgement (black arrows) and air bronchograms bilaterally (white arrows) COVID-19: coronavirus disease 2019; CT: computed tomography; RT-PCR: reverse transcription-polymerase chain reaction

**Figure 4:**
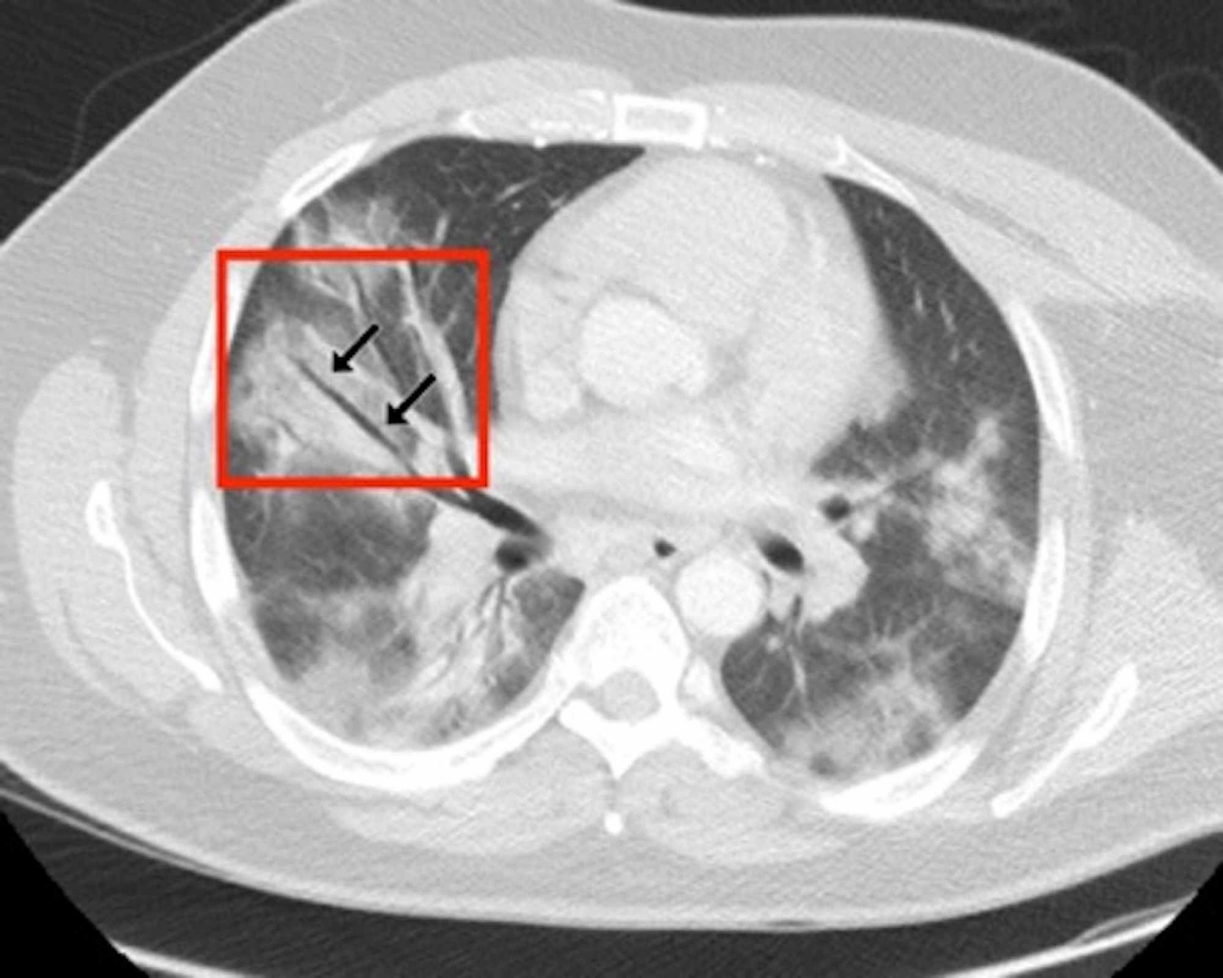
Axial CT through lung bases of a male patient (lung parenchymal window level settings) A 45-year-old male patient presented with cough, fever, and progressive shortness of breath while self-isolating. His respiratory function deteriorated and he was admitted to the hospital. A chest CT was performed, which showed evidence of COVID-19 and subsequent RT-PCR was positive. An axial contrast-enhanced CT image of the chest showed bilateral patchy ground-glass and consolidative changes with areas of confluence. There was a linear air bronchogram seen in the right upper lobe (black arrows). This was surrounded by peri-bronchial consolidation and ground-glass changes COVID-19: coronavirus disease 2019; CT: computed tomography; RT-PCR: reverse transcription-polymerase chain reaction

**Figure 5:**
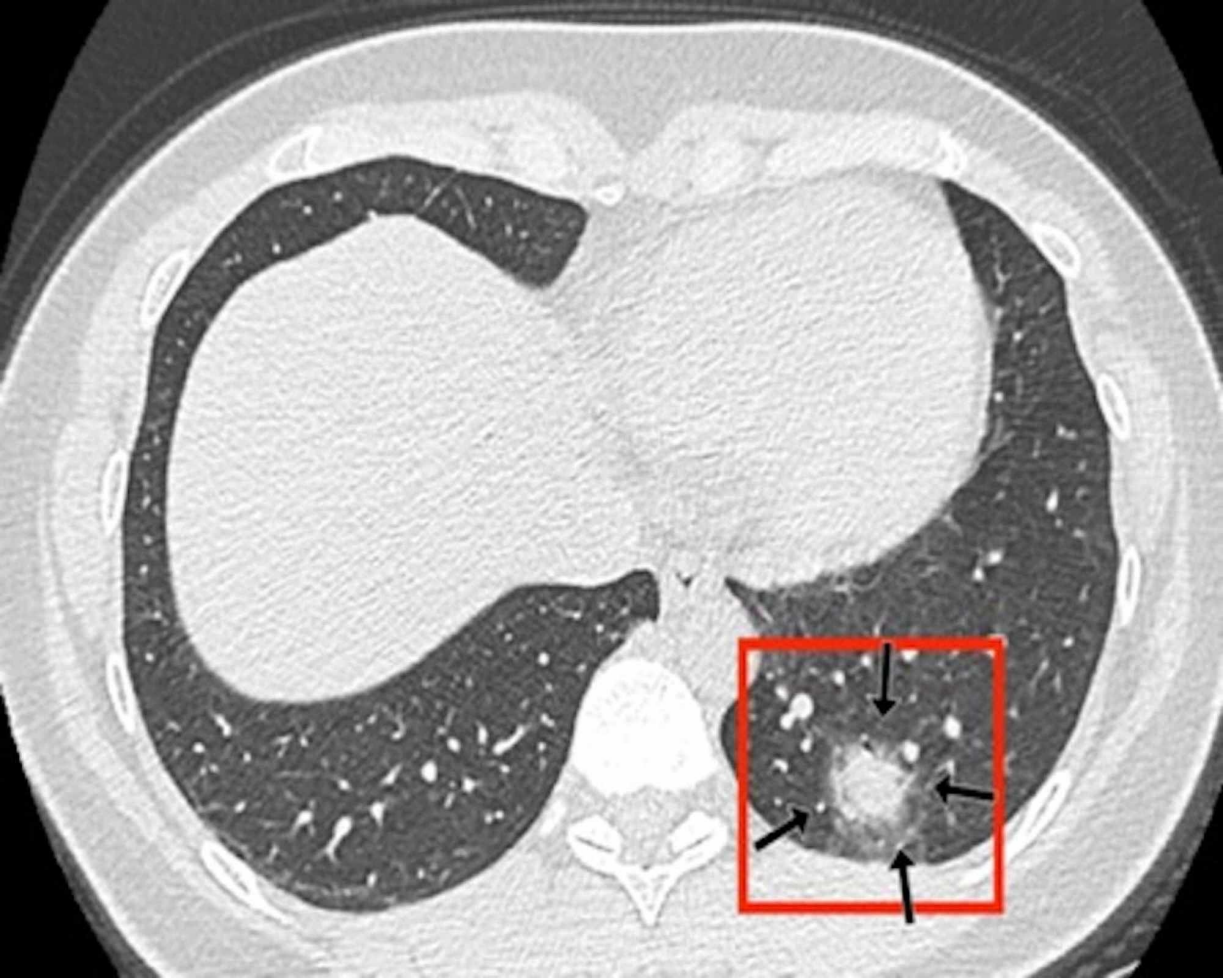
CT halo sign A 27-year-old female patient presented with cough, loin to groin pain, and fever. A CT of the abdomen was performed to look for renal calculi. However, in view of her cough, her chest was also scanned for evidence of COVID-19. An axial non-contrast chest CT image demonstrated an example of a halo sign. There was a discrete focal consolidation within the left lower lobe as outlined in the red box. The nodule showed a peripheral rim of ground-glass opacification, halo sign (black arrows) COVID-19: coronavirus disease 2019; CT: computed tomography

**Figure 6:**
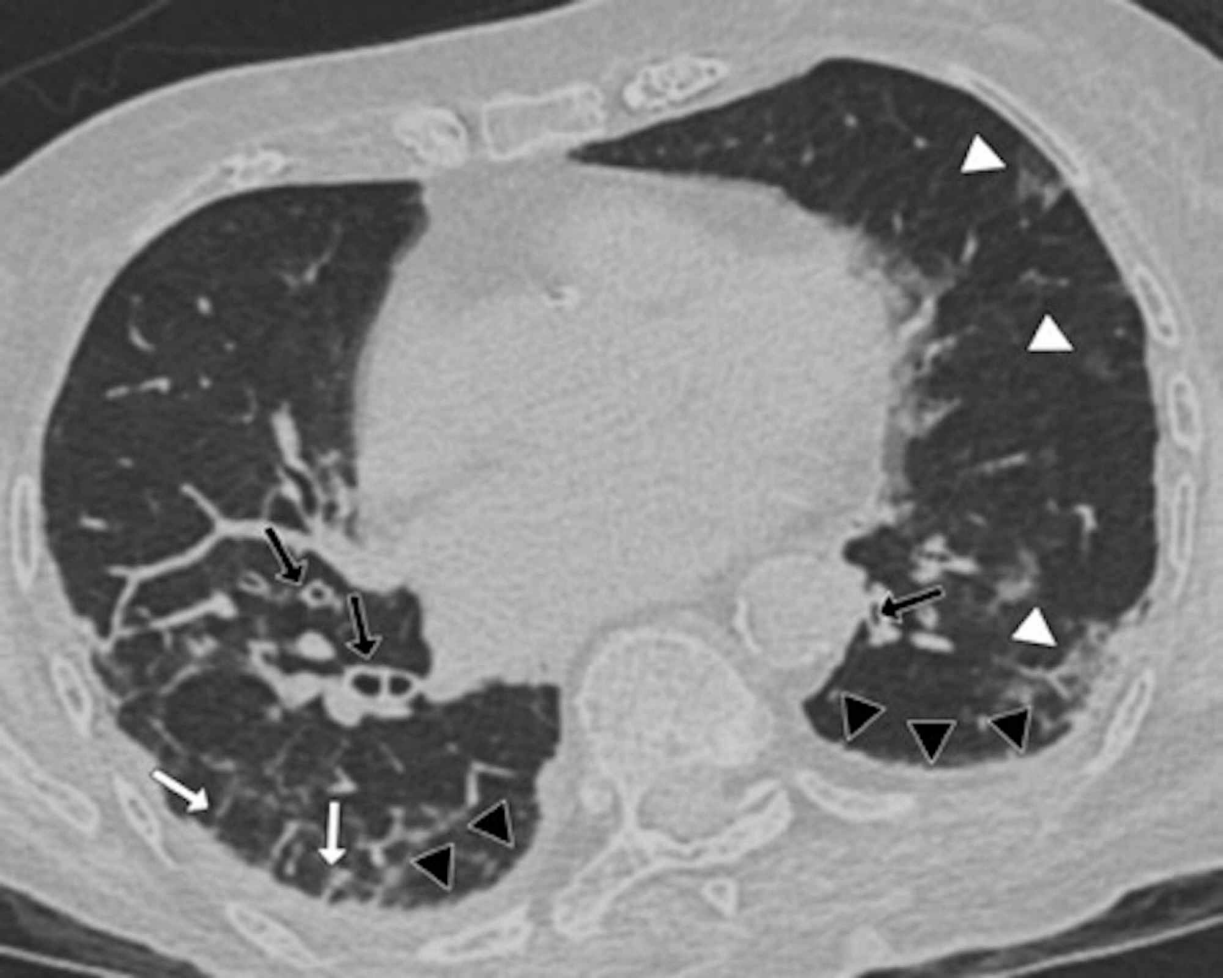
Interstitial and parenchymal findings An 88-year-old female patient presented with progressive respiratory distress and equivocal X-ray findings. A chest CT was performed for diagnostic purposes and the subsequent RT-PCR result was positive for COVID-19. An axial unenhanced CT image showed evidence of right bibasal septal thickening (white arrows) with small patchy areas of ground-glass consolidation (white arrowheads). This patient also demonstrated bilateral bronchial wall thickening (black arrows), an unusual finding in our cohort. In addition, there was bilateral basal pleural thickening and shallow pleural effusions (black arrowheads) COVID-19: coronavirus disease 2019; CT: computed tomography; RT-PCR: reverse transcription-polymerase chain reaction

**Figure 7:**
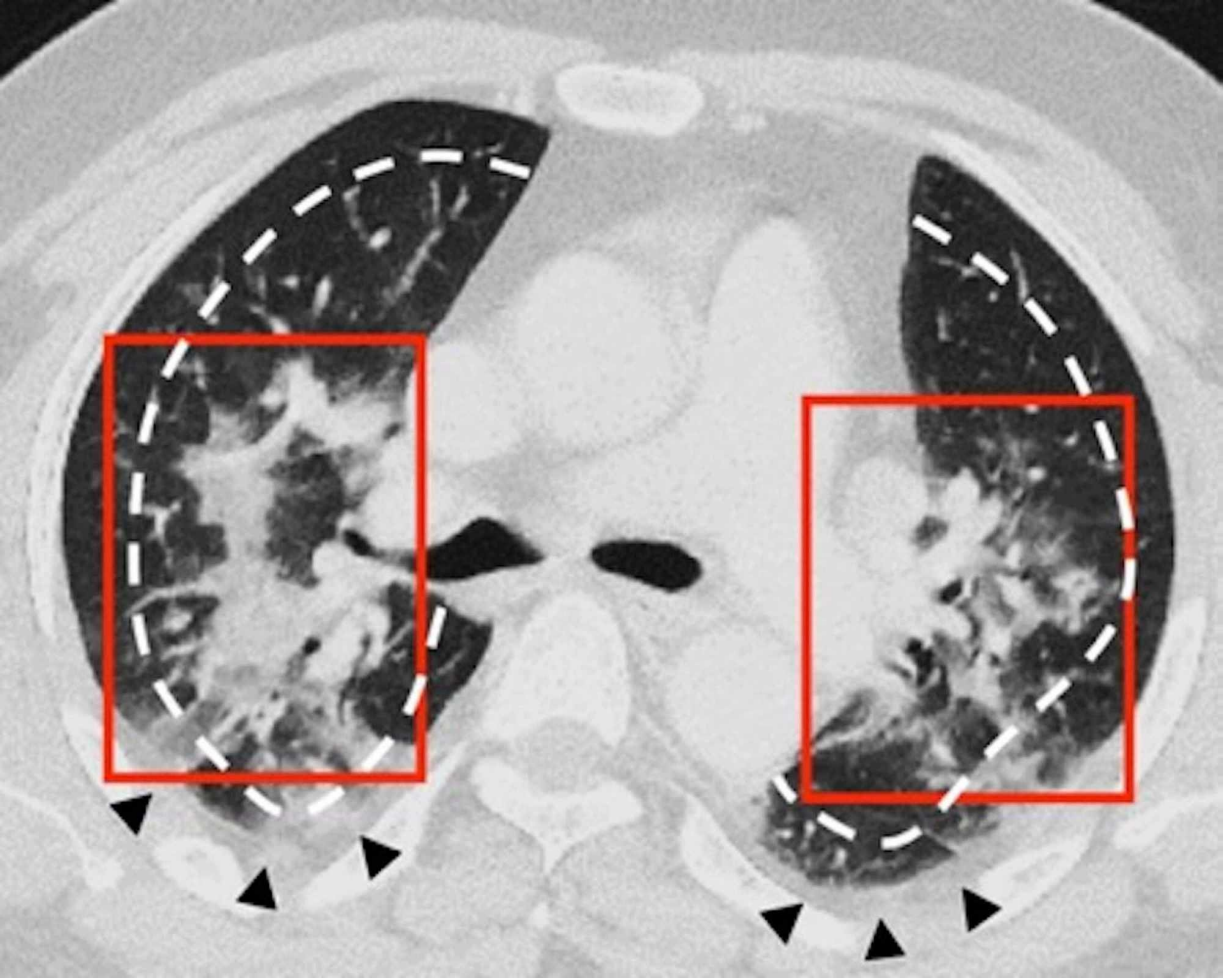
Disease distribution on axial plane: central and peripheral A 53-year-old male patient presented with left-sided abdominal pain and raised inflammatory markers. A CT of the chest was performed for COVID-19 screening. His RT-PCR returned a positive result. An axial contrast-enhanced CT of the chest showed bilateral COVID-19 lesions in a central distribution (red boxes). There were patchy areas of ground glass and consolidative changes with areas of confluence. The lesions were predominantly in a perihilar location. The central distribution was demarcated by the white dashed lines, which divided the lungs according to the inner two-thirds and outer one-third. Bilateral pleural thickening was also present (black arrowheads) COVID-19: coronavirus disease 2019; CT: computed tomography; RT-PCR: reverse transcription-polymerase chain reaction

**Figure 8:**
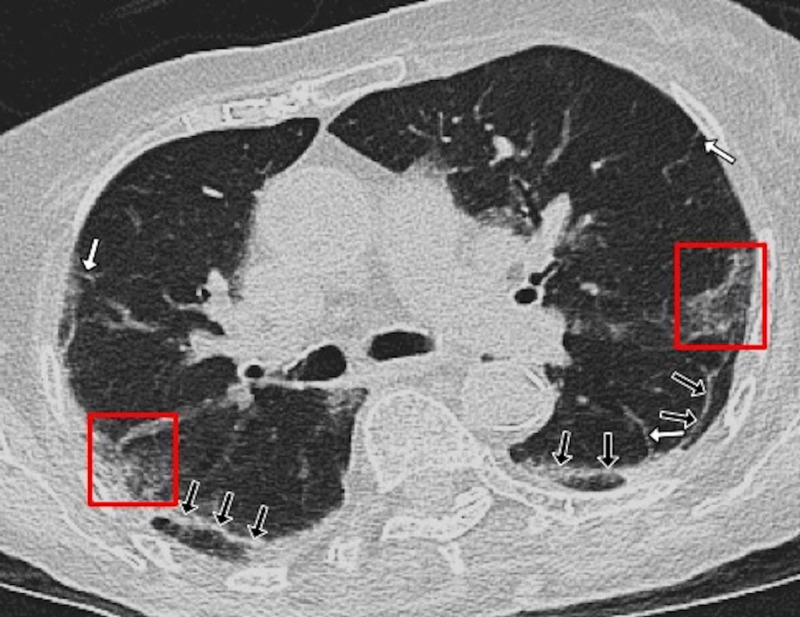
A 67-year-old female patient with positive RT-PCR test for COVID-19 The patient's CT images showed a subpleural band, a common finding in our cohort of patients. An axial contrast-enhanced CT image demonstrated a thin curvilinear subpleural band seen posteriorly in both lower lobes (black arrows). In addition, there were patches of peripheral ground-glass opacification (red boxes) and thickened interlobular septa (white arrows) COVID-19: coronavirus disease 2019; CT: computed tomography; RT-PCR: reverse transcription-polymerase chain reaction

Patients’ CT features are presented in detail in Figures 3, 4, 5, 6, 7, 8. A majority of cases (56%) had moderate severity scores. There was no correlation between the severity score and mortality, p=0.790 (Table 4).

**Table 3:** Chest CT lesion characteristics and severity scores CT: computed tomography

Predominant density	Number of cases	Percentage
Ground-glass opacity	8	44%
Consolidation	7	39%
Mixed	3	17%
Bilateral	18	100%
Unilateral		0%
Predominant shape		
Amorphous	13	72%
Rounded	5	28%
Patchy/nodular lesions	11	61%
Pattern morphology and other associated findings		
Crazy paving	4	22%
Mosaic pattern	4	22%
Tree-in-bud	1	6%
Halo sign	7	39%
Reverse halo sign	-	0%
Pleural thickening	9	50%
Pleural effusion	7	39%
Fibrosis	3	17%
Mediastinal lymphadenopathy	3	17%
Subpleural fibrotic line	11	61%
Subpleural sparing	6	33%
Focal vascular engorgement	15	83%
Bronchial wall thickening	2	11%
Septal thickening	13	72%
Air bronchograms	6	33%
Well-defined	10	56%
Ill-defined	6	33%
Both	2	11%
Lobar involvement (case bases)		
Right upper lobe	17	94%
Right middle lobe	16	88%
Right lower lobe	18	100%
Left upper lobe	15	83%
Left lower lobe	18	100%
Most severely involved lobe (severity score 3 or 4)		
Right upper lobe	3	17%
Right middle lobe	2	11%
Right lower lobe	5	28%
Left upper lobe	4	22%
Left lower lobe	4	22%
Severity score		
Mild (0–6)	6	33%
Moderate (7–13)	10	56%
Severe (14–20)	2	11%
Central	2	11%
Peripheral	14	78%
No predilection	2	11%
Anterior	1	6%
Posterior	14	78%
No predilection	3	17%

**Table 4:** Relationship between CT severity score and mortality CT: computed tomography

Severity score	Alive		Dead		Total
	N	Percentage	N	Percentage	
Mild	5	83.30%	1	16.70%	6
Moderate	7	70.00%	3	30.00%	10
Severe	1	50.00%	1	50.00%	2
Total	13	72.20%	5	27.80%	18
Fisher’s exact test:					
P=0.790					

## Discussion

The severe acute respiratory syndrome coronavirus 2 (SARS-CoV-2), the pathogen for COVID-19, is an animal-to-human and human-to-human transmissible and potentially lethal virus that belongs to the coronavirus viridine family. Its clinic-radiological lung manifestations strongly differ between Chinese, Korean, and Italian populations. To the best of our knowledge, this is the first preliminary report describing the chest CT features of COVID-19 in a cohort from London, UK.

In our patients, the initial symptoms were similar to those described in the existing literature, including cough, fever, and shortness of breath. However, two out of 18 patients (11%) in our cohort presented with abdominal pain and one (6%) with vomiting. CRP levels were high in all cases, which is consistent with previous studies [12-15]. In the early stage of COVID-19 pneumonia, CRP levels could be positively correlated with lung lesion sizes. CRP levels could reflect disease severity and could be used as a key indicator of disease prognosis [15]. D-dimer levels were measured in five cases in our cohort [16]. Four out of five tests were positive. All five cases underwent CTPA with negative results for PE. PE rates have previously been reported to be around 20% [17-18], which contrasted with our findings with no PE encountered. Lymphopenia is one of the prominent features of COVID-19. Lymphocyte counts may be a useful biomarker in predicting the severity of the disease [19]. Lymphocyte counts were low (56%) or normal (44%) in all cases in our patient cohort.

Lung manifestations of COVID-19 are similar to those of SARS and MERS. Radiological studies have shown cardinal similarities, including bilateral patchy GGO and consolidations, subpleural involvement, and predominant posterior and peripheral lesion distributions [2,4-6,8-13,20,21]. The CT findings of COVID-19 pneumonia in the UK have been generally consistent with those in the Far East [20,22]. However, the proportion of predominantly consolidative lesions in a Chinese cohort was found to be approximately 30-60% [22,23]. In Korean patients, no predominantly consolidative lesions were observed, which is consistent with our study findings [2]. In addition, the proportion of chest radiographic abnormalities was 60% in Chinese COVID-19 patients [24], but 33% in Korean patients [2]. Considering these radiologic observations and five deaths from COVID-19 in the UK, UK patients seem to experience a harsher disease course than Korean patients. We did not observe the reversed halo signs in our cohort, but a few cases with the reversed halo sign were observed in some other recent reports [2,24-26]. Septal thickening, a subpleural fibrotic line, and focal vascular engorgements were seen frequently in our study.

This study was performed at one of the epicenters of the COVID-19 pandemic, in London, UK. Our hospital has received a high number of COVID-19 patients, but CT scanning has been reserved for equivocal chest X-Ray findings, excluding complications like PE and preoperative patients scanning to exclude COVID-19 before the RT-PCR test result. Therefore, the initial recommendation in our institution, as in several other UK hospitals, was to perform CT-CAP in all preoperative patients. However, this recommendation was changed in May 2020. Currently, only limited sections through both lung bases appear sufficient to detect COVID-19 lung pneumonia for patients who undergo semi-elective or urgent surgery [27].

According to a statement that was recently published by the British Society of Thoracic Imaging, the assumed role of CT in the diagnosis, triage, and prognosis of patients with COVID-19 infection continues to be refined, and is reserved for clinically and laboratory-suspected COVID-19 patients who are seriously ill with oxygen saturation of less than 94% or a National Early Warning Score (NEWS) equal to or greater than 3. Normal or equivocal chest X-ray and CT examinations can be carried out with or without contrast as in our study [28]. Furthermore, in line with the British Society of Thoracic Imaging guidelines, patients with persistent significant abnormalities on the second chest X-ray, abnormal pulmonary function tests, and/or significant unexplained breathlessness may require further investigations, which can include a pre-contrast high-resolution volumetric CT and CTPA to assess the presence of interstitial lung disease and PE, respectively [29].

There are several limitations to our current study. Firstly, it was a retrospective single-center study with a limited patient cohort without follow-up CT examinations. Secondly, CT examinations were carried out with and without contrast enhancement, but we did not face great difficulties to see the lung lesions, and we clearly observed vascular engorgement signs on post-IV contrast-enhanced CT examinations. Thirdly, the RT-PCR test was only performed twice in our institute. And lastly, the time interval between initial symptom onset and CT was not precisely defined, but RT-PCR and chest X-ray were performed on the patient admission date, and subsequent CT scans were performed either on the same day or within a week’s time, with or without RT-PCR test results.

## Conclusions

Chest CT scanning was not a first-line investigation modality for suspected COVID-19 patients; all patients had initial chest X-ray at the time of admission along with an RT-PCR nasopharyngeal swab test. Mixed GGO-consolidation, amorphous morphology, and posterior and peripheral lung distributions of the COVID-19 pneumonia lesions were the cardinal findings in our limited but diverse UK patient cohort. Subpleural sparing, bilateral pleural effusion, presence of a halo sign, subpleural fibrotic lines, septal thickening, focal vascular engorgements, mediastinal lymphadenopathy, absence of a reverse halo sign, and absence of cavitation were observed in our study. Subpleural sparing, which has never been reported in the literature in English, was observed in our cohort. A majority of the UK cases presented with cardinal symptoms including fever, shortness of breath, and cough. All patients’ CRP levels were elevated. Lymphopenia and normal lymphocyte counts were observed in our patient cohort. All D-dimer-positive cases did not show any PE, which has never been reported in the literature in English.

The findings from our limited cohort are generally in line with CT imaging findings from the published literature worldwide with a few exceptions, such as the presence of pleural effusion, mediastinal lymphadenopathy, and the lack of reverse halo sign.
